# Locating Victimhood: Sexual Violence and Institutional Gaslighting in the British Military

**DOI:** 10.1177/10778012261440223

**Published:** 2026-04-07

**Authors:** Hannah K. Richards

**Affiliations:** 1 1980 Cardiff University, Cardiff, UK; 2 School for Policy Studies, University of Bristol, Bristol, UK

**Keywords:** British armed forces, sexual violence, military justice, institutional gaslighting, military violence

## Abstract

This article examines how the British military responds to sexual violence experienced by its servicewomen. Drawing on observations of 15 hearings at a British military court center, it argues that the logic of operational effectiveness saturates the material and conceptual foundations of military justice. The article shows how the institution is imagined as the victimized subject within the military courts, in turn creating the conditions for the institutional gaslighting of the victim-survivors of sexual violence. This article is the first in-depth, qualitative study of the British military courts, and provides an important new empirical contribution to the study of sexual violence within militaries.

## Introduction

Militaries across the world are variously reckoning with the issue of sexual violence perpetrated by those within their ranks. Scandals have erupted in France, Canada, Australia, the USA, and the United Kingdom (UK), each triggering sustained periods of public scrutiny followed by institutional announcements of new “zero-tolerance” policies, promises of reform, and commitments to better support the victims of sexual violence (Baron, 2024; Bonnes, 2024; Herriott et al., 2023; MacKenzie, 2023). In the UK, military personnel are prohibited from speaking publicly on defense matters without authorization and researchers face well documented barriers to engagement with the institution and its personnel (Catignani & Basham, 2021; Gray et al., 2023). As a result, knowledge about the nature and extent of sexual violence within the British military—as well as the efficacy of the institutional steps taken to address these behaviors—is partial and contested. Thus, questions around how sexual violence is understood and responded to within the British military, and the implications of this on the daily lives of military personnel, are left largely unanswered (cf. Gray et al., 2023).

Drawing on observations of hearings held at a British military court center between 2021 and 2023, this article responds to these questions by shedding new light on the day-to-day application of military justice in sexual violence cases. Bringing to the fore the micro-politics of a military court center, the article demonstrates what sustained, feminist-informed attention to the in-situ workings of one of the mechanisms used to respond to sexual violence can reveal about how violence against servicewomen is conceived of, and managed by, the institution. In particular, I ask who are the imagined subjects of military justice and what are the implications of this for how sexual violence is prosecuted in the military? Through my consideration of the spatio-temporal dynamics of a military court center, I explore how specific conceptions of sexual violence and the victimized subject are produced and justified within this space. In so doing, I demonstrate how military justice mechanisms can create the conditions for the institutional gaslighting of servicewomen who experience sexual violence.

Thus far, scholarship on the relationship between military justice and sexual violence has focused on the competing legal frameworks that govern military personnel (Hillman, 1999; White, 2023), assessments of justice system performance (Carpenter, 2023; Eichler, 2021; Herriott et al., 2023), media coverage of rape and sexual assault trials (MacKenzie, 2023), and how those subject to sexual violence experience the reporting, investigation, and prosecution of such offences (Bonnes, 2024; Gill & Febbraro, 2013; Gray et al., 2023). Within this research, however, military justice is primarily discussed as something disembodied, faceless, and placeless (c.f., Tosto & Bonnes, 2022). This article seeks to rectify this omission by populating and animating the otherwise neglected spaces of military justice to reveal the politics and assumptions built into their daily operations. Moreover, in turning to examples of military misconduct that take place in more everyday settings away from operational contexts, the article challenges the tendency within the literature on military justice to focus on violence perpetrated *by* the British Military rather than violence taking place *within* it.

I begin the article by providing important background information about the origins and purpose of the British military's justice system, focusing in particular on the debate surrounding jurisdiction for the investigation and prosecution of sexual violence in a military context. Although this literature is limited in its discussion on the micro-politics of military justice, it nonetheless provides important context for the subsequent analysis. In the next section, I outline the conceptual framework that guides this research. I draw on feminist theories of gaslighting (Berenstain, 2020; MacKenzie, 2023; Ruíz, 2020; Sweet, 2019) to show how institutional responses to sexual violence can create conditions for further harm. Following this, I introduce data from my fieldwork to explore three key processes through which the conditions for institutional gaslighting are made possible within the military court center. First, I show how totalizing and exclusionary conceptions of military work are baked into the materiality of the space of the court center in ways that deny the realities of sexual violence within the military. Second, I explore how the concept of “operational effectiveness,” rather than justice, shapes the daily procedures of sexual violence hearings at the courts, severing this violence from structures of power within the British military that produce it (Berenstain, 2020). Finally, I consider how sexual violence policy measures are constituted as a threat to an otherwise cohesive and efficient system of military justice, allowing for the institution to be figured as a central victim of such violence. In focusing on the spatial and temporal dynamics of the court center, I provide a novel conceptual and empirical contribution to the study of sexual violence within militaries, revealing how, in its own responses to sexual violence, the institution is figured as the victimized subject of such violence, rather than the women who experience it.

### Military Justice and Sexual Violence

There is a well-established, dedicated system of justice that governs the British armed forces. In its present form, the British military's system of justice (the Service Justice System or SJS), broadly mirrors that of the civilian Criminal Justice System (CJS). The SJS has its own prosecuting authority, court service, military police, sentencing guidelines, complaints system, and detention center (see Blackett & Reed, 2023).^1^ The existence of a separate system of military justice is not distinct to the British context, and has existed in some form for centuries (Duxbury & Groves, 2016; White, 2023).

The rationale for maintaining a distinct justice system is often presented as self-evident: military work is unique and violent. Four main arguments are often put forward to justify the existence of the SJS: First, a timely and efficient justice system is presented as essential to supporting the operational effectiveness and morale of armed forces within liberal democracies (Blackett & Reed, 2023, p. 10). Second, military justice is considered central to the maintenance of discipline within the military. Third, the SJS reflects the “special and unique nature of the Armed Forces” (Blackett & Reed, 2023, p. 10), ensuring that military personnel accused of disciplinary or criminal offences will be judged by those who understand the pressures of military work (Blackett & Reed, 2023, p. 10). Fourth, the SJS is portable, it extends English and Welsh law to personnel serving overseas who, in this context, would ordinarily be outside the jurisdiction of domestic civilian courts.

There is a rich and important body of work—spanning the disciplines of Military History, Law, and International Relations—that explores military justice in relation to known violence committed by British military personnel on overseas operations and in colonial territories (Bennett, 2014; Kemp, 2019; Williams, 2018). Whilst this research makes a valuable contribution to the discussion of military justice and state accountability, it focuses on violence perpetrated *by* the British Military rather than violence taking place *within* it. Yet, the privileging of operational or conflict contexts ultimately foregrounds an imagination of the institution in which proximity to highly destructive violence is understood as *the* defining character of military life (Basham, 2015; MacKenzie, 2023). This imagination obscures the mundanity, banality, and boredom that can actually characterize daily military work, disconnecting it from the violence enacted by the institution (Basham, 2015; MacKenzie, 2023).

In this way, it is the *framing* of military service as unique that plays a central role in enabling and justifying a separate system of justice. For MacKenzie, within public imaginations of the institution, military work is framed, more than other workplaces, “as a form of disciplined sacrifice that is extreme and worthy of reverence” (MacKenzie, 2023, p. 9). Here, the concept of military exceptionalism (MacKenzie, 2023) is particularly helpful as it captures how:

Many workplaces—such as hospital emergency rooms—might be described as extraordinary or extreme; however, there seems to be a more widely accepted understanding that national militaries are more exceptional in ways that distinguish them from other workplaces. (MacKenzie, 2023, p. 20)

The concern here is not whether militaries *are* indeed unique employers, but rather *how* they are described in ways that separates them and distinguishes them as “in a category of their own” (2023, p.21). Central to this process of distinction, are the myths of the “good institution” and the “good soldier,”^2^ “which are constructed as necessarily white, masculine, exclusive, and reproduced through the regulation of sex and the exclusion of women and racialized groups” (MacKenzie, 2023, p. 9; see also Basham, 2013; Bonnes, 2020). Indeed, when instances of misconduct away from the battlefield emerge, these myths enable such violence to be framed as a natural byproduct of institutional and soldier dedication rather than something produced through systemic dysfunction (MacKenzie, 2023, pp. 25–26). For example, reports of violent, misogynistic behavior have been excused as necessary for unit bonding, or the result of the exhaustion and pressure following a deployment. Such imaginaries thus enable these institutions to be understood not only as special, but also not accountable to civilian standards and laws (MacKenzie, 2023, p. 20; see also Hillman, 1999; Razack, 2004). This framing expects deference to soldiers, while also lessening or diluting the harm inflicted by them.

However, recent public scrutiny of military justice systems has called into question the ability of these mechanisms to effectively investigate sexual offences. For example, in 2024 the Canadian parliament introduced legislation to strip the military of its powers to investigate and prosecute all sexual offences committed by its personnel domestically, handing these powers instead to the civilian justice system (Brewster, 2024). In contrast, in 2023 the US Department of Defense announced that cases of sexual assault would remain within the military's justice system but would no longer be prosecuted within the accused's chain of command (US Department of Defense, 2023). In the UK, two relatively recent independent reviews of the military's justice system recommended that cases of rape and serious sexual assault (committed in the UK) be taken out of the military's jurisdiction (Atherton, 2021; Lyons, 2020). Initially the Ministry of Defence (MoD) rejected these recommendations (MoD, 2020), but some concessions have since been made. For example, in 2023, the civilian prosecution service announced that it would exercise concurrent jurisdiction with the military for sexual offences, including rape and sexual assault (Crown Prosecution Service, 2023). Yet, it remains unclear how decisions over jurisdiction are reached, with the latest official statistics indicating that numerous cases of sexual violence are still being dealt with in the military system (MoD, 2025). These examples of the differing approaches taken toward the jurisdiction of sexual violence in a military setting allude to the substantial political contestation over the “ownership” of such violence that currently accompanies the discussion of a global military #MeToo movement (Bourke, 2021).

In the UK, official statistics show that in 2024, British military police forces initiated 341 investigations into sexual offence allegations (not including investigations into historic offences), 297 suspects were men, whereas 289 victims were women (MoD, 2025). This figure, however, is thought to represent only the tip of the iceberg: according to a survey administered in the same year, “13% of all female personnel report being subject to sexual harassment in a Service environment, whilst for male personnel, this figure is less than 1%.” (MoD, 2024, p.15). Similarly, data from interviews with veterans (Edwards & Wright, 2019; Gray et al., 2023), internal reviews (MoD, 2019; MoD, 2020), and parliamentary inquiries (Atherton, 2021) further indicate that a significant number of servicewomen experience some form of sexual violence during their careers. The evidence also suggests that incidents of sexual violence often go unreported due to concerns and confusion about the reporting processes, the stigma of reporting, fears of retaliation and the perceived impunity of the perpetrators (Gray et al., 2023; Herriott et al., 2023).

Past scrutiny of the British military's responses to sexual offending suggests that the system is characterized by low conviction rates (Herriott et al., 2023), a lack of support provided to those who have experienced sexual violence (Gray et al., 2023), and significant failures in the investigation of such offences (Centre for Military Justice, 2023). Indeed, various scholars have highlighted how the social organization of military life—particularly through its privileging of warrior masculinity and military brotherhood—fosters a culture conducive to the perpetration of violence across intimate and global scales (Basham, 2013; Bonnes, 2020; Wadham & Connor, 2023). As such, this raises the question of whether a justice system that is rooted in an “underlying hypermasculine military culture, which is seemingly permissive of many of the risk factors associated with sexual violence perpetration” (Herriott et al., 2023, p. 13) can ever legitimately or appropriately respond to cases of sexual violence.

Yet, those who defend the military's continued investigation and prosecution of sexual violence do so with reference not only to the distinct nature of military work, but also to the substantial backlogs and delays faced within the civilian CJS (HM Crown Prosecution Service Inspectorate, 2024). These arguments emphasize that “it is likely that there would be even fewer convictions” (Blackett & Reed, 2023, p. 27) if military sexual offences were to move into the CJS, something especially pronounced following the delays and backlogs caused by the COVID-19 pandemic. Hence, both justice systems—military and civilian—have logistical and structural issues that present obstacles to the future of successful sexual violence prosecutions in the UK. Whether sexual violence cases “belong” in a military system of justice (or should be subsumed into the civilian system) is not the central question of this article. Instead, I examine what the in-situ workings of one of the military's primary mechanisms used to respond to sexual offending can tell us about how gendered violence is conceived of and responded to in the military courts.

### Institutional Gaslighting and the Military

The dynamics of the court center discussed later in this article could be read through multiple theoretical lenses. For example, I could have drawn attention to the interconnectedness of the structural and the intimate violences of the courtroom, highlighting how they exist on a continuum of violence, troubling the binaries of war/peace and private/public (Cockburn, 2004; True, 2020; Wibben, 2019). Similarly, the concept of secondary victimization (Carroll, 2022; Madigan & Gamble, 1991; Pemberton & Mulder, 2023), could have also provided a useful tool to unpick the ways in which servicewomen may be further harmed through the processes of military justice. Yet, in this article I read the material through the lens of institutional gaslighting. As will become apparent, the concept of institutional gaslighting is particularly helpful for drawing attention to how institutions play a central role in sustaining existing power structures and to the work that goes into denying and resisting critique about the harmful effects of such structures (MacKenzie, 2023, p. 28).

Traditionally, the term gaslighting has been used in the fields of psychiatry and psychology to describe a form of emotional abuse within interpersonal relationships in which a perpetrator convinces his—it is most commonly a man perpetrating this abuse against a woman—target that she is epistemically incompetent, distorting her sense of reality (Barton & Whitehead, 1969; Klein et al., 2023; Stark, 2019; Stern, 2007). Yet, the centering of interpersonal and psychological dynamics can work to obscure the “*social* characteristics that give gaslighting its power” (Sweet, 2019, p. 852). Indeed, gaslighting cannot exist as something separate from the social world and, consequently, the existing inequities and power imbalances through which it is constituted.

In treating gaslighting as a purely psychological phenomenon, it is often assumed that once women report their experiences of coercive control that they will be safe in legal and mental health systems. However, using the case of domestic violence, sociologist Paige Sweet shows how gaslighting both draws from and exacerbates gender-based power imbalances that are already present in intimate relationships *and* in the larger social context (Sweet, 2019, p. 856). Accordingly, Sweet's research shows that even in supposedly “safe” legal or health systems, intersecting stereotypes around the irrationality of women or the aggressiveness of Black women, for example, persist, rendering institutions such as the police and the courts as critical sites for further gaslighting, to the extent that these institutions can be considered as “unknowing colluders” (Sweet, 2019, p. 867) in the gaslighting process.

In this context, although institutions may be a *feature* of abuse, they themselves are not figured as the abuser. However, scholars looking at medicine (Carter, 2022; Ruíz, 2020), the law (Chapman, 2022; Epstein & Goodman, 2019), and education (Dunn, 2023; Gildersleeve et al., 2011) have applied the concept of gaslighting to contexts beyond interpersonal relationships to show how institutions themselves create the conditions for the gaslighting of minoritized groups such as women, in particular, Black and Indigenous women. Through intangible obstacles—such as credibility discounts in the courtroom^3^—and more tangible ones—such as the prohibitive cost of healthcare (Carter, 2022)—these institutions employ tactics that deny and trivialize the realities of minoritized groups in ways that sustain existing raced, sexed, and gendered power relations. Important here then, is that these institutions are explicitly connected to broader unequal power structures.

Although the concepts of institutional betrayal (Gray et al., 2023; Monteith et al., 2021) and institutional abuse (Wadham & Connor, 2023) have variously been employed to capture the multifarious harms produced by military institutions, MacKenzie (2023) is the first to apply the concept of institutional gaslighting explicitly in relation to the military. In her study of the public rhetoric that surrounds sexual violence in the military (in the USA, Canada, and Australia), Mackenzie identifies three gaslighting techniques adopted by military institutions (MacKenzie, 2023, p. 29). First, those outside of the military (for example, researchers and charity workers) are feminized, rendering them inexpert, and unable to generate legitimate critique of the military institution. Second, the narratives surrounding sexual violence in the military convey a message that the problem is solved, in turn inferring that incidents of sexual violence in the military are an isolated, rather than systemic problem. Third, the rhetoric acknowledges that there may be a problem with sexual violence in the military but presents solutions that exclude and deny the experiences of those who have been subjected to such violence. These gaslighting techniques, MacKenzie shows, are only effective when connected to the well-established myths that surround Western militaries such as a belief in their innate goodness and exceptionalism reproducing existing structural inequalities (MacKenzie, 2023).

Importantly, MacKenzie also acknowledges the significant role that Black and Indigenous feminist scholarship has played in highlighting that gaslighting is not related to a perpetrators’ goals or intentions, “but the function of its operation” (Berenstain, 2020, p. 735; see also Ruíz, 2020). Indeed, even when individuals, communities, or institutions consider themselves to be “well-intentioned” or “allies” (McKinnon, 2017) or if they engage in active or willful ignorance (Mills, 2007), they are still positioned to produce harm. What is more, ignorance and allyship turn attention to the intentions of the more powerful actors, rather than the harms produced by them, thus obscuring the ways in which structural gaslighting “draws its power from and simultaneously reinforces structural oppression in an unending positive feedback loop” (Berenstain, 2020, p. 735).

Having established that institutions themselves can create the conditions for the gaslighting of minoritized groups, in this article I examine the micro-politics of a military court center to explore how violence against servicewomen is conceived of, and managed by, the institution. In particular, I ask who are the imagined subjects of military justice and what are the implications of this for how sexual violence is prosecuted in the military?

## Methods: Conducting Courtroom Observations

Although academic research acknowledges the social and political contestation over the boundaries of military justice, the military and its laws are primarily discussed as something disembodied, placeless, and faceless, with the military courts “rarely seen or heard” (Grady, 2016, p. 714). As such, the data collected for this project focused on locating, populating, and animating the military court center to examine how responses to sexual violence are enacted in the everyday workings of one of the mechanisms tasked with tackling it. In what follows, I outline the methods used during this project, highlighting the particular ethical challenges of doing this research.

Between December 2021 and July 2023, I conducted observations of 15 hearings held at one of the UK's permanent military court centers. Much like in civilian criminal proceedings in the UK, Military court center hearings are open to the public and thus provide a unique opportunity to observe how conditions for harm are produced and sustained through mundane, everyday practices. Within socio-legal research, court observations are a well-established, but time-consuming method and the depth and quantity of “data” available to the researcher within a single courtroom setting is well documented (Roach Anleu et al., 2015). Although there are two permanent military court centers, I chose to conduct observations at only one of them, Bulford Military Court Center (hereafter Bulford). I prioritized gaining in-depth familiarity with one, already complex, location over seeking breadth or comparison between different court centers. In total, I spent over 150 hours at the court center and produced approximately 500 pages of fieldnotes. I began observations as soon as restrictions from the COVID-19 pandemic began to ease and members of the public were again allowed back into the court center as observers. To determine a feasible sample, I drew on my initial literature review, early visits to the court center, and an awareness of the emotional impact of observing trials to determine when to stop collecting data for this project (Smith, 2020).

The broader project from which this article stems was focused on how violence (not limited to sexual offending) *within* the military was understood in relation to the violence exerted *by* the military. The hearings were sampled opportunistically. Although court listings were uploaded online, they only outlined the date and time of the hearings, providing little other information. As such, my decision about which hearings to observe was based primarily on my availability to turn up to the court center in person and to ask the administrative staff the nature of the hearings taking place (Smith, 2020). Of the 15 hearings I observed, eight involved charges of sexual violence that ranged from sexual assault to rape, the other seven involved charges of fighting, battery, going absent without leave, and disobeying a lawful command. All the charges related to allegations taking place in non-combat contexts. In the sexual violence hearings I observed, the defendants were all white men, and the claimants all white women. The below findings and discussion section begins by drawing on data produced from the observation of all 15 hearings before turning to the eight sexual offence hearings in more detail.

The court center itself was often busy, and I took various practical steps to ensure that I was as transparent as possible in all my interactions (Smith, 2020). For example, I carried several copies of the participant information sheet with me in the court center, allowing me to respond quickly to questions about my presence and to provide my contact details to those interested in learning more about the project (Smith, 2020). In addition, I was provided with a visitor's badge for each visit which I clearly displayed alongside my University ID card. Although seemingly insignificant, these visual signifiers of my exteriority did routinely remind individuals of my presence. These various measures reflect the idea that obtaining consent during observational research is “an on-going process that requires an active reflexivity” (Plankey-Videla, 2012, p. 1) on the part of the researcher.

In contrast, in relation to the witness testimony, particularly that of the victim-survivors, consent proved much more difficult to navigate. Firstly, Smith (2020) highlights the potential for harm in seeking informed consent from witnesses prior to a hearing as this may cause them “unnecessary extra consideration during [the] trial” that may lead to the witness feeling “uncomfortable, embarrassed, or distracted by the presence of a researcher” (Smith, 2020, pp. 11–15). In this light, I made the decision not to obtain verbal or written consent from witnesses prior to the hearings. Before each witness gave testimony, the presiding judge would however, remind them of the public nature of the hearing, making clear that journalists and other members of the public may be sitting in the public gallery. While there was no obvious or perfect solution, it nonetheless felt important to include perspectives that had been thus far written out of academic research on the SJS (Gray et al., 2023).

As with any ethnographically informed research, the analysis of my data began long before my handwritten notes were neatly typed up into a word document. Indeed, how I understood my interactions “in the moment” at the court center; the decisions I made around what to scribble down in a notebook when there; how I chose to type these notes up later; and subsequently what I chose to present in this article, all constitute some form of “analysis” (Faria et al., 2020, p. 1103). In the UK, the digital recording of court hearings is generally prohibited, consequently socio-legal scholars have long acknowledged that, “it is impossible to capture everything occurring in [the] natural setting” (Roach Anleu et al., 2015, p. 386) of a courtroom. As such, following Olivia Smith (2020) I produced a template that would document the presenting demographic details of the trial participants, the charges against a defendant, the Judge's remarks, and summaries of the key arguments made by the defense and prosecution.

Yet, as highlighted by Sharyn Roach Anleu and colleagues, “the flow” of courtroom dynamics remained “better conveyed in terms of a narrative rather than recorded on the pre-printed template” (2015, p. 380). Thus, in addition to populating the template, the fieldnotes themselves focused on the content, structure, and delivery of the defense and prosecution cases (for example, who the central characters were and how they acted in the courtroom, what timeline of events was presented, why participants were said to have acted in such a way). The intention was thus not to produce a verbatim transcript of the words spoken during the trial, but rather to consider how those within the courtroom were invited to make sense of these words through the social and cultural resources available to them.

Consistent with interpretivist approaches to research, in addition to this “in the moment” analysis, I subsequently conducted careful (re)readings of my fieldnotes from my observations (Kurowska & de Guevara, 2020; Yanow, 2007). My reading approach to these notes was informed by Laura Shepherd's (2021) narrative analytical strategy. Such a strategy pays attention to the stories through which meaning is conveyed to an audience, and how a “particular construction of self/ selves (subjects) and surrounding objects with which those subjects are held in relation” (Shepherd, 2021, p. 24). Shepherd advocates for a double-reading of texts that first focuses on mapping the narratives that exist in a given text. I thus began by establishing what stories were being told about sexual violence by actors within the court center and how these stories were constructed, paying particular attention to their chronologies and characters (Shepherd, 2021). Following Shepherd, the second close reading of my fieldnotes considered how the narratives identified worked to *produce* meaning within the court center and was thus informed by discourse theory. Here, discourse is understood not simply as “language” or “talk,” but rather as the “systems of meaning-production […] that fix meaning, however temporarily, and enable us to make sense of the world” (Shepherd, 2008, p. 20). Discourses work to configure attachments of meaning to objects and subjects, and the relations between them, thus constituting the “raw material of narrative” (Shepherd, 2021, p. 31).

In deconstructing particular discourses, researchers work to show how “subjects and objects emerge as known/knowable” and how these discourses create “relational chains of meaning between these subjects and objects such that they are known/ knowable in particular ways— according to particular logics” (Shepherd, 2021, p. 32). In particular, I paid close attention to the implicit and explicit relations of “*opposition*, *identity*, *similarity*, and *complementarity*” (Doty, 1993, p. 306) that worked to afford particular meanings to the narratives within the courtroom. This second reading was thus iterative and involved continuously “tacking back and forth between the theoretical and the empirical” (Kurowska & de Guevara, 2020, p. 1215). In adopting this narrative analytical strategy, I was able to ask not only *who* the imagined subjects of military justice are, but also to consider the implications of this for how sexual violence is prosecuted in the military.

## Findings and Discussion

### Building a World of Exceptionalism in the Military Courts

In the early stages of my research, I had (incorrectly) thought of the military court center simply as one of the fixed, physical sites that provided a material home to the object of my study, military violence, and not as an *agent* in its own right (Jeffrey, 2019; Massey, 1994; Mulcahy, 2011). Yet, in reflecting on how both myself and others interacted with not only the physical space, but also the imagined spaces of military justice, it became clear that the spaces within and of the court center were actively *doing* something. For example, the court center itself is nestled on the outskirts of a military base. It sits behind a barbed wire fence, there is an empty guardroom, and it is variously populated with uniformed military personnel. Yet as you enter the site, there is a sign reminding visitors of the slogan of the Military Court Service; ‘independent and impartial’. Similarly, in email correspondence before my first visit, I was reminded that the court was open to the public and that *any* day that it was sitting I would be able to “turn up and watch proceedings.” Hence, despite the Centre being labelled as a site of open justice and one that was professedly independent from the military institution, it is simultaneously presented as somewhere that is distinctly military in nature. Even upon entry to the site, it is clear that the center is characterized by uneasy tensions between ideas of open, independent justice *and* institutional proximity. These tensions already begin to confuse and distort the supposedly clear boundaries placed around military justice for those entering this space.

This is something further emphasized by the naming of the two courtrooms at Bulford. Above the signs for “Courtroom 1” and “Courtroom 2” there are gilded letters that denote other names for these spaces; Waterloo and Minden.^4^ In naming the two courtrooms after battles of near mythological status within the British military, the space behind the court doors is directly, and transparently, linked to two geographically and temporally disparate theatres of battle that conjure nostalgic imaginaries of the collective might and battlefield heroism of the British armed forces. The naming of rooms, buildings, and ships in this vein is a common feature of British military spaces. Yet, in the context of the court center, rather than evidencing the independence of the legal spaces, it again suggests instead that they are deeply imbricated within specific understandings of military history and military work.

Although “Waterloo” and “Minden” *do* provide space for hearings of charges that are distinct to the military—for example, going absent without leave or disobeying a lawful command—they also hold stories of much more recognizable offenses to a civilian observer such as sexual assault, rape, and battery. In all the hearings I observed, the offences were alleged to have taken place in the more mundane contexts of bedrooms, bars, health centers, offices, and swimming pools. Yet, what merits attention when entering “Waterloo” or “Minden” is the question of how the hearings fit into a broader tapestry of military discipline, history, and innate “goodness.”

After sorting out my notes. I stand up and take a look at the artwork down this end of the corridor. In addition to the painting and photograph of the submarine, there is also a painting entitled ‘The Irish Guards enter Pristina’. Next to this is a colour photograph of two, white MTP-wearing^5^ soldiers with camo paint and rifles, stealthily placed in long grass […] A bit further down the corridor, there is another piece of art […] In small print it says, “Lockheed Martin Orlando f1, Richard Thompson 1997”. I can’t find a copy of it online. (fieldnotes January 2023).^6^

Similarly, the above extract draws attention to how the corridors of the court center were also lined with a variety of different images, ranging from photographs of camouflaged soldiers on training exercises, to portraits of monarchs, and regimental paintings that conveyed chaotic yet heroic front-line operations. The protagonists in these visual representations are invariably white men. Much like the names of the courtrooms, the images of military life in the communal areas of the court center thus work to foreground the idea that a white man's proximity to noble forms of highly destructive violence is the defining character of military life (see MacKenzie, 2023, p. 21).

Significantly, as Elena Ruíz shows, structural gaslighting can have an epistemic, world building function that produces “totalizing and abusive ambients—languages, stories, buildings, practices, rituals, forms, and documents” (Ruíz, 2020, p. 696) that work to destroy resistance to dominant narrations of power and deny competing claims to authority. Here, the simple act of designating the courtrooms and corridors as belonging to a broader, heroic military history powerfully reinforces popularly held assumptions about the benevolence and, ultimately, the naturalness of British military violence. The naming of these spaces thus works to obfuscate and deny how military violence is also sustained by and productive of harms to gendered, raced and classed “others,” (see Basham et al. 2024) others that are notably absent from this space. Even before entering the courtroom then, the innocence and well-meaning intentions of the British military are established as natural and unquestionable, laying foundations for the exoneration of the institution from discussion of how it may be implicated in producing cultures that are permissive of sexual violence (Berenstain, 2020; Ruíz, 2020).

The material indicators of the institution's exceptionalism outlined above help bring to life something that had been inferred across many of the trials I observed. On numerous occasions, purely through their location in the exceptional realm of the military, soldiers were assumed to be innately “good” unless proven otherwise. Indeed, in several of the sexual assault trials, the character evidence put forward by the defense barristers variously referred to the defendant's “reputation for professionalism and diligence [that] has followed him throughout his career” (Fieldnotes May 2022), his ability to work “as part of a close-knit team of both males and females” (Fieldnotes May 2022), or that he was a “man of good character and clearly a very capable soldier” (Fieldnotes June 2022). Underpinning, these accounts is an assumption that being a good soldier is interchangeable with being of good character (in a legal sense).

Yet, it was only in the final trial (for charges of rape and sexual assault) that I observed that this assumption was explicitly articulated. When discussing the defendant's character evidence, the judge explained that,

“[t]his is why it is a court martial and not in the civilian court. The Board^7^ will be used to dealing with soldiers, they will see that this is a junior NCO [non-commissioned officer], who no doubt will have good character references”

and that,

“because most people who appear in these courts *are* of exceptionally good character, because otherwise they would have been dismissed and would be appearing in the Crown Court.” (Fieldnotes March 2023)

Here the “soldier” is established as something inherently different to a civilian public. Clear lines are thus drawn around good/bad and civilian/military in ways that invoke the myths of the “chivalrous, self-sacrificing, and responsibly masculine” (Basham, 2018, p. 36) institution.^8^

Importantly, this assumption of innate goodness is presented as an objective legal truth within the courtroom; to question this is to question the authority of the court. For example, during a sentencing hearing for a case of rape and sexual assault, a statement was read aloud to the court in which the victim (also a soldier) described how she felt “angry that the Army doesn’t feel the need to properly address these types of behaviour” (fieldnotes June 2023). The defense barrister challenged the inclusion of this sentence, arguing that the statement had not been disclosed to him in advance and, “so far as there is criticism of the wider Army… that is not something to be laid at [the perpetrator's] feet,” to which the judge responded “and it won’t be. He is being sentenced only by failings of his own, not anyone else” (fieldnotes June 2023). The victim's attempt to connect instances of sexual violence to broader institutional cultures is figured instead as an unfair and unsubstantiated allegation, one that can be easily disregarded as overly emotional or irrelevant to the hearing.

As with other hearings I observed, patterns of sexist, misogynistic, and racist behavior that extended beyond the actions of an individual perpetrator were deemed to be outside of the court's remit. The constitution of the court as a seemingly pure legal arena extracts the trial participants from their broader social context, suggesting that their experiences cannot be understood as indicative of a systemic problem, but rather an isolated incident already being addressed (MacKenzie, 2023; Williams, 2018). The siloing of the courtroom as an objective legal arena, alongside the material evidence of the “goodness” of the institution and its personnel thus work in tandem to create conditions that are conducive to the institutional gaslighting of victims of sexual violence. During my observations, there was no denial that sexual violence may take place within the institution, yet the accounts of sexual violence given were partial and limited, denying and erasing “experiences that could challenge carefully crafted images of the good military and good soldiers” (MacKenzie, 2023, p. 29). Sexual violence is thus acknowledged as a problem, however the dominant narratives deployed in this space can simultaneously work to render the experiences of those impacted by this behavior irrelevant (MacKenzie, 2023, p. 28).

### The Guiding Logic of Operational Effectiveness

During my observations, the narratives deployed in the courtroom routinely alluded to operational effectiveness, unit cohesion, and overseas deployment during trials for sexual assaults that had allegedly taken place in the more mundane moments of service life in the UK. For example, during one hearing (a sexual assault case), extracts from the 2018 sentencing guidelines issued by the Office of the Judge Advocate General (OJAG) were read aloud to the court. This included the statement that, “minor sexual assaults are more serious in a Service context than in civilian life because they can cause resentment and undermine unit cohesion” (fieldnotes July 2022).^9^ This was echoed a year later, in a sentencing hearing of a soldier who had been found guilty of the rape and sexual assault of his colleague. When sentencing the soldier, the judge reminded the court that,

“Service personnel have little choice where and with whom they serve”, often “accommodated with only curtains or, when on operations, nothing separating them” … they may share facilities, they work, eat, socialise and sleep together …“sexual offending undermines the bond of trust that must exist… affects morale and ultimately operational effectiveness…” (Fieldnotes July 2023)

Despite the offences being ones where women were subject to sexual violence in everyday contexts in the UK (an office or a bedroom), having no obvious military quality or connection, these statements nevertheless remind those in the court of the ever-present threat of war. In so doing, they serve as a reminder that the individuals occupying this space are of interest to and an intrinsic part of the broader military machinery.

Consequently, this narrative suggests that the harms of such violence can also be felt by the institution itself. It is not that the impact of sexual violence upon the women that experienced these offences was ignored, indeed as detailed earlier, victim impact statements documenting, amongst other things, anger and betrayal, were read aloud to the court. Yet, during the sentencing for criminal offences in the military courts, guidance from the civilian Sentencing Council is applied concurrently with that provided by the OJAG, quoted above. When assessing the harm of criminal conduct, the OJAG guidance requires the court to consider “service factors which may increase harm.” These include a consideration of whether the offence has had an adverse effect on either the: operational effectiveness, and/or morale, unit cohesion or discipline, and/or on the reputation of His Majesty's Armed Forces, and whether there is a disparity in rank (Office of the Judge Advocate General, 2025, p. 60). This additional guidance thus produces alternative narratives about the harms and impact of sexual violence within the courtroom, turning attention away from the claimant and positioning the institution instead as the central victim of such behavior.

[The defendant moves behind the privacy screen at the back of the court as the other usher makes his way over to the door by the prosecution corridor. […] A young white woman enters the room, she is wearing her Army issue khaki jacket and skirt with a cream shirt and pale green tie]

**Judge:** Good morning, Private Wilson.^10^ Please salute the Board.

[She looks confused and doesn’t salute. The judge motions towards the Board and repeats the request. She moves slightly and then salutes. Given that we have been made aware that she might have trouble giving evidence, it seems like a strange formality […] After this, the usher talks her through the affirmation. She stumbles a little bit over it. Two women came in with her: the first seems to be witness support (a white woman in civilian clothes wearing a lanyard and ID card) and the second is a white female Army officer]

**Judge:** I would say try to relax, “but it is a really difficult chair to sit in” (Fieldnotes December 2022)

The above excerpt is taken from another sexual assault hearing, in which the claimant, Private (Pte) Wilson, had requested special measures in court.^11^ Despite the recognition of her vulnerability or potential for intimidation, military court procedures dictate that Pte Wilson's first action upon entry to the courtroom is nevertheless to stand and salute the senior officers (four men and one woman) who form the Board. Thus, despite being identified as a vulnerable, alleged victim of a sexual assault within the courtroom, Pte Wilson is still expected to display deference to the senior ranks in the room and to identify herself first and foremost in relation to her position within the military. In so doing, the court is again reminded that the needs of the institution, over those of the individual, come first. Although a momentary performance, in centering her identity as a soldier, the court is encouraged to see the harms allegedly perpetrated against Pte Wilson, as ones of institutional import.

In foregrounding ideas of operational effectiveness and soldiering identities, the sexual violence hearings are thus figured as being deeply entangled with the functioning of the institution. Importantly, this entanglement enables the military and its justice system to be established as *the* legitimate authority in constructing knowledge about and responses to the sexual violence perpetrated by those within its ranks. This, in turn, constitutes the intimate, embodied knowledge of these issues that is held by women both within and outside of the institution as epistemically less valuable. Hence, this claim of authority can be used to undermine or discredit alternative accounts of sexual violence in the military, including those put forward by the victims of such violence in the courtroom and those researching this topic.

### The Threat of Political Interference

At Bulford, it is not sexual violence alone that is presented as a threat to the cohesion and efficacy of the institution, indeed during my research it became apparent that the policy measures adopted to address such misconduct also constituted a threat to the military.

From the early days of my fieldwork, it was made clear to me that sexual assault hearings were relatively common in the court center. Conversations about the frequency of sexual violence cases seen in the courts were sometimes accompanied by expressions of frustration (“I thought the culture might have changed between generations”), sadness (“the sad thing is, there are hundreds of cases like this”), and at times, even tedium (“it is exciting when it is something a bit different”). My interlocutors provided several reasons about why there might be so many sexual offence cases making their way to the courts: for example, a backlog of cases from COVID-19, increasing awareness of how to report such offences, and the excessive alcohol consumption that characterizes military social events. Underpinning many of these conversations was the inference that the need to tackle sexual offending in the military was becoming imperative and that, given the external scrutiny the SJS was facing, it was something that could no longer be ignored. Within these discussions, the institution's relationship with sexual violence was figured as one of imminent reckoning, in which the ordinarily self-autonomous world of military justice was colliding with the destabilizing force of external scrutiny. Much like the foregrounding of operational effectiveness and soldiering identities, such conversations worked to position the institution and its justice system at the center of discussions around the impacts and harms of sexual violence within the military.

While conducting observations, I watched as various measures—such as a new “zero-tolerance” policy, or the requirement for at least one woman to be on a Board—moved from political rhetoric into everyday features of the court. People spoke freely (both in open court and in corridor conversations) about these changes and, when discussing the sexual violence trials I had been observing, these measures were routinely provided as evidence of the SJS's commitment to protecting servicewomen, and of how an evolving SJS remained an appropriate setting for dealing with sexual violence cases. Interestingly, one of the sexual assault trials that I observed took place eight weeks after the formal publication of the MoD's “zero-tolerance” policy. The trial involved allegations that a senior soldier within a unit had sexually assaulted a younger, more junior colleague on two occasions. He was found guilty of one charge of sexual assault.

**Prosecutor:** I don’t wish to do this whilst the defendant is present. But it would be helpful to have an indication of whether sentencing will happen this week.

**Defense barrister**: I’m not sure a pre-sentencing report is necessary unless, and I’d be surprised, if you had dangerousness in mind.

**Judge**: I wouldn’t be having in mind dangerousness […] You’ll be aware of current armed forces policies and attitudes towards offences of this nature.

**Defense barrister**: I’d want a suspended… but given the current climate … I’d think custodial. (Fieldnotes May 2022)

During both the trial and sentencing hearing, the new sexual misconduct policy measures were positioned as marking a significant departure from how things had previously been done in the court center. In the above extract, the legal counsel and judge are discussing the next steps after the guilty verdict had been returned by the Board. In this exchange, as with many others during the trial, those imbued with legal authority in the courtroom worked together to navigate the seemingly ambiguous parameters and expectations of the “current climate” in which they were operating. There was acknowledgement that whilst they clearly did not conceive of the defendant as “dangerous” (a view also supported by the prosecutor) the new expectations of how this type of offence should be managed might mean that he may have to be dealt with as such (a custodial sentence, for example). Here then, we see a collision between how things would have been dealt with in “normal times”—the times before the zero-tolerance policy—and the uncertainty and ambiguity of these “current times.”

This uncertainty was carried through to the later stages of the discussion as shown in the below excerpt.

**Judge:** So, in this instance, it is dismissal rather than reduction of rank. Now, there is an added complication of the new armed forces policy. The new policy dictates that offences of this nature will result in immediate dismissal […] Because this court has more powers over employment than a civilian court […]

**Defense barrister:** The difficulty is that this new policy, it usurps the discretion of the court […] The reality is that if this had been in a Crown Court, it is very unlikely that we would be looking at custody […] Even for a reputable individual, it is his first offence […] We know of the considerably positive aspects of the defendant's private life. […]

**Judge**: Is there a case for finding out what the unit intends?

**Defense barrister**: Once the chain of command is engaged –

**Judge:**—yes, this is something supervened by the policy. The policy on retention was tight before, it is now zero-tolerance. But it is unclear whether it is to be applied retroactively…

**Defense barrister**: The unit may look favourably on retention, he has done a very specific role for them…

[I note down that there is a lot of trailing off, and references to ‘these times’]

**Judge:** It would be quite unusual, but let's see what the unit intends.

**Prosecutor:** The policy has shifted the ground somewhat when it comes to sentencing. (Fieldnotes May 2022)

In this discussion between the judge, prosecutor, and defense barrister, careful attention is paid to whether the policy could (and should) be applied to cases that had taken place before the policy's introduction. Thus, the new policy, whilst seemingly providing clear guidance about the threshold of dismissal, was simultaneously presented as temporally ambiguous. It is associated with ideas of uncertainty and ambiguity that originate from outside the court and that threaten to “usurp” its authority. The zero-tolerance policy was introduced as part of a set of measures to “enable services to better support victims and secure justice for them” (MoD, 2022) yet here we see its application being reframed in ways that draw attention to its impacts on more powerful actors within the court center.

Despite the confusion around the zero-tolerance policy, a sentence of dismissal was ultimately decided upon in accordance with the new developments, and the wider debate around the policy was deemed “not a matter for these courts” (fieldnotes July 2022). In locating policy discussions as belonging outside (both temporally and spatially) this specific legal arena, the court's impartiality and independence is again established. In this light, the court's authority, and its position as a natural, objective, and trusted provider of justice, is reaffirmed. Positioned simultaneously as a temporal “other” *and* a powerful external force, the zero-tolerance policy is thus presented as an external interruption to the “normal” times of military justice, a political intervention in an otherwise “apolitical” space.

The positioning of external scrutiny and policy measures in such a manner has significant consequences for how sexual violence and those who have experienced this phenomenon are conceived of within the courts. First, the allusion to external interference in an otherwise objective space places the issue of sexual violence firmly within a separate “political sphere.” When contrasted to the impartiality, rationality, and objectivity of the court, the “political” nature of this topic infers that the policy measures are the product of a temporary, even faddish, agenda. Consequently, stricter sanctions for sexual violence can be figured as over the top or excessive, particularly if perpetrators are not deemed to be dangerous by the legal parties within the courtroom. This again works to establish the military and its justice system as *the* legitimate authority in constructing knowledge about and responses to sexual violence.

Second, political interference is constituted as having an impact on the normal functioning of military justice. When considered alongside the benevolence of the institution discussed previously, the military and its courts, rather than the servicewomen subject to sexual violence, are imagined as the victimized subject. Consequently, the harms associated with sexual violence are rendered intelligible through their relation to the institution, rather than the servicewomen subject to such violence. In each of these considerations, the institution itself is extracted from any complicity in facilitating the conditions in which sexual violence can take place and is instead figured as another victim of such violence (MacKenzie, 2023; Nayak, 2009).

## Conclusion

This article has provided a new empirical contribution to the study of sexual violence within militaries. Through my consideration of the spatio-temporal dynamics of a British military court center, I reveal how, in its own responses to sexual violence, the institution is figured as the victimized subject of such behavior, rather than the women who experience it. In demonstrating how the daily operation of the court center works to deny and obscure the realities of how sexual violence may play out in the everyday lives of military personnel, I argue that the military courts create conditions for the institutional gaslighting of servicewomen who experience sexual violence.

Within the court center, understandings of sexual violence are oriented around the perceived impact it will have upon the cohesiveness and effectiveness of the institution. Through the logic of operational effectiveness, sexual violence hearings are reframed in a way that extricates the institution from any accusations of complicity, enabling it to be figured as another potential victim of such violence (see MacKenzie, 2023; Nayak, 2009). In so doing, sexual violence is severed from the structures of power that may give rise to it in the first place (Berenstain, 2020), such as the gendered power inequalities that exist within the institution. Moreover, the adoption of new sexual violence policy measures is also constituted as a threat to an otherwise cohesive and efficient system of military justice, allowing for the institution to be figured as a central victim of such violence.

Overall, rather than protecting military personnel, I argue that military justice can expose and reproduce other, less visible forms of harm. To make claims that it takes these violences seriously, the military must recognize that the experiences of servicewomen may not always conform to the image of the institution it imagines itself to be. Dominant ideas of military exceptionalism work to deny the realities of servicewomen, obscuring the fact that good soldiers too are capable of perpetrating sexual violence against their colleagues. Without starting from this acknowledgement, institutional responses to sexual violence are unlikely to deliver the accountability and safety that victims deserve.

## Notes

1. The Service Prosecuting Authority and the Military Court Service are independent from the Ministry of Defence and the three Services, although they are variously staffed by military personnel.

2. I use the word ‘soldier’ throughout this article as a general reference term for all members of the British military, including those who serve(d) in the Army, Royal Navy, and Royal Air Force. This is done to aid the comprehensibility and readability of the text. At times, I also make a distinction between officers and ‘other ranks’. There are two distinct tiers within the British armed forces. Officers have higher authority than the ‘other ranks’, having entered their respective service after a period of officer training. In contrast, the ‘other ranks’ (which constitute the majority of military personnel), enter their service after a shorter period of training and have less authority. Throughout their career, a soldier can work their way up through the ‘other ranks’, eventually becoming a ‘Non-Commissioned Officer’.

3. Credibility discounts refer to the gender-based myths and narratives the circulate within the law that work to discount the credibility of women in this system. These include rape myths and myths about the irrationality and emotionality of women (Epstein & Goodman, 2019; Tuerkheimer, 2021).

4. The Battle of Waterloo (1815) saw the Duke of Wellington’s army (alongside the Prussian Forces) defeat Napoleonic French forces, ending the violent upheavals that had been taking place across Europe since the French Revolution. The Battle of Minden (1759) was one of the greatest Allied victories of The Seven Years War in which the smaller Anglo-Hanoverian forces defeated the larger French Army.

5. MTP stands for multi-terrain pattern and is the standard camouflage of the British armed forces.

6. When quoting fieldnote extracts throughout this article, the text that appears within double quotation marks indicates that it is an exact quote. Speech that is not in quotation marks is my summary of what was said in note form and thus cannot be read as an exact quotation. Text in italics denotes that it is my own personal observations or reflections.

7. A Board is the equivalent of a jury in civilian courts. A Board is composed of three or six military ‘lay’ members—depending on the severity of the offence—who are always of a more senior rank than the defendant. Toward the end of my fieldwork (in January 2023) alterations were being made by the Military Court Service to ensure that at least one woman and one man were present on each Board. More recently, mixed-Service Boards have also been adopted.

8. This is echoed beyond the walls of the court center. For example, in a judge-led review, when discussing the comparatively low conviction rates for sexual offences in the SJS, the report notes that, “[a]ll defendants at Court Martial are likely to be of Good Character in the legal sense (as of course will be the complainant) and the Judge will give a Good Character direction. In CJS [civilian Criminal Justice System] rape trials many defendants will be of Good Character but not to the extent that exists in the SJS.” (Lyons & Murphy, 2020, p. 47)

9. This phrasing has been removed from the latest version (2025) of the sentencing guidelines.

10. All trial participants have been given pseudonyms or generic titles, such as ‘judge’ or ‘defense barrister’.

11. Special measures, such as the use of screens or video-recorded evidence, for example, are used in both the civilian and military courts when witnesses are considered vulnerable or intimated (Crown Prosecution Service (CPS), 2022)
